# Sertoli cell‐derived exosomal MicroRNA‐486‐5p regulates differentiation of spermatogonial stem cell through PTEN in mice

**DOI:** 10.1111/jcmm.16347

**Published:** 2021-02-19

**Authors:** Quan Li, Hanhao Li, Jinlian Liang, Jiaxin Mei, Zhen Cao, Lei Zhang, Jiao Luo, Yan Tang, Rufei Huang, Huan Xia, Qihao Zhang, Qi Xiang, Yan Yang, Yadong Huang

**Affiliations:** ^1^ Department of Cell Biology & Guangdong Provincial Key Laboratory of Bioengineering Medicine Jinan University Guangzhou China; ^2^ Department of Pharmacology Jinan University Guangzhou China; ^3^ Guangdong Provincial Institute of Biological Products and Materia Medica Guangzhou China; ^4^ Institute for Translational Medicine Shenzhen Second People’s Hospital / The First Affiliated Hospital of Shenzhen University Health Science Center Shenzhen China; ^5^ Guangdong Provincial Biotechnology Drug & Engineering Technology Research Center Guangzhou China; ^6^ Biopharmaceutical Research & Development Center of Jinan University Guangzhou China

**Keywords:** differentiation, exosome, miR‐486‐5p, Sertoli cells, spermatogonial stem cells

## Abstract

Self‐renewal and differentiation of spermatogonial stem cell (SSC) are critical for male fertility and reproduction, both of which are highly regulated by testicular microenvironment. Exosomal miRNAs have emerged as new components in intercellular communication. However, their roles in the differentiation of SSC remain unclear. Here, we observed miR‐486‐5p enriched in Sertoli cell and Sertoli cell‐derived exosomes. The exosomes mediate the transfer of miR‐486‐5p from Sertoli cells to SSCs. Exosomes release miR‐486‐5p, thus up‐regulate expression of *Stra8* (stimulated by retinoic acid 8) and promote differentiation of SSC. And PTEN was identified as a target of miR‐486‐5p. Overexpression of miR‐486‐5p in SSCs down‐regulates PTEN expression, which up‐regulates the expression of STRA8 and SYCP3, promotes SSCs differentiation. In addition, blocking the exosome‐mediated transfer of miR‐486‐5p inhibits differentiation of SSC. Our findings demonstrate that miR‐486‐5p acts as a communication molecule between Sertoli cells and SSCs in modulating differentiation of SSCs. This provides a new insight on molecular mechanisms that regulates SSC differentiation and a basis for the diagnosis, treatment, and prevention of male infertility.

## 1 INTRODUCTION

Self‐renewal and differentiation of Spermatogonial stem cells (SSCs) are the foundation for spermatogenesis. A delicate balance exists between self‐renewal‐commitment and differentiation of SSC. Excessive self‐renewal or differentiation of SSCs hinders spermatogenesis hence inducing male infertility. SSC is supported within niches (specialized microenvironments) which provide various endocrine and paracrine signals for regulating self‐renewal and differentiation of SSC.^1^, ^2^

In mammalian testes, Sertoli and Leydig cells are the primary contributors to the SSC niches. Glial cell line‐derived neurotrophic factor (GDNF), secreted by Sertoli cells, is a growth factor that was first identified as a crucial self‐renewal factor of SSCs,^3^ which is indispensable for self‐renewal of SSC. Fibroblast growth factor 2 (FGF2), also secreted by Sertoli cell, is another growth factor essential for the self‐renewal of SSCs.^4^, ^5^ Besides, Leydig cells produce cytokine colony‐stimulating factor‐1 (CSF‐1) that regulates the self‐renewal of SSC and guides SSCs to their final position.^6^ Regarding the differentiation of SSCs, it is well established that testosterone is a primary regulator of spermatogenesis.^7^ Sertoli cell‐secreted retinoic acid (RA), an active metabolite of vitamin A, induces meiotic entry of spermatogonia.^8^ Also, activin A and bone morphogenic protein 4 (BMP4) enhances differentiation of SSC.^9^, ^10^ Nonetheless, factors and molecular mechanisms in differentiation of SSCs are largely unknown.

Exosomes are nanometre‐sized (50‐100 nm) vesicles. Increasing evidence showed that exosomes were detected in different body fluids, participate in intercellular communication through incorporation of their cargo into the target cells.^11^ Various cells can release miRNAs via exosomes which function in a paracrine manner in the surrounding microenvironment, and promote cell development.^12^ MSC exosomes mediate cartilage repair by promoting proliferation and attenuating apoptosis.^13^ Fibroblast derived exosomes promote epithelial cell proliferation through the TGF‐β2 signalling pathway.^14^ Additionally, exosomes could mediate cardiac regeneration, which highlights the potential utility of exosomes as cell‐free therapeutic candidates.^15^ In our study, we observed Sertoli cell‐derived exosomes (SC‐EXO) promoted SSCs differentiation compared with Leydig cell‐derived exosomes (LC‐EXO). To obtain a better understanding of how SC‐EXO regulate SSCs differentiation, we used a miRNA array and bioinformatics to analyse the miRNA landscape of SC‐EXO and LC‐EXO. And the top 10 miRNAs were analysed, among which miR‐486‐5p was found to positively regulate SSCs differentiation. miR‐486‐5p has been proven to regulate stem cell development and differentiation. Overexpression of miR‐486‐5p induces a premature phenotype and inhibits proliferation of MSCs, whereas inhibition of miR486‐5p has the opposite effects.^16^ Additionally, miR‐486‐5p promotes mammary gland development and location.^17^ However, the role of exosome‐derived miR‐486‐5p in SSCs differentiation remains largely unknown.

Here, we attempted to characterize the molecular mechanisms of miR‐486‐5p in SSCs differentiation. Results suggested that miR‐486‐enriched exosomes could transfer from Sertoli cells to SSCs and promote SSCs differentiation by targeting PTEN. Our findings revealed a mechanism in regulating the differentiation of SSC that might have an implication in male infertility.

## 2 MATERIAL AND METHODS

### 2.1 Animals

Mice used in experiments were purchased from the Experimental Animal Center of Guangdong Province, China. Animals were maintained under a 12 hours light/dark cycle and a controlled temperature (24 ± 2°C) with relative humidity (50%–60%). The standard rodent diet and drinking water were freely accessible. All experiments were conducted according to the National Institute of Health guidelines for the care and use of animals and approved by the Institutional Animal Care and Use Committee of Jinan University.

### 2.2 Spermatogonial stem cell (SSC) isolation and culture

Spermatogonial stem cell derived from testes of six‐day‐old male mice. To obtain SSCs suspension from the testicular tissue, a two‐step enzymatic digestion protocol was applied.^18^ Briefly, decapsulated testes were treated with collagenase type IV (1 mg/mL) for 15 minutes at 37℃, followed by digestion in 0.25% trypsin and 1 mmol/L EDTA for 10 minutes at 37°C. The singly dissociated cells were incubated overnight in dished to removed somatic cells. Non‐adherent and weakly adherent cells were collected and then labelled these cells with CD326 (EpCAM) MicroBeads by Mini MACS Starting Kit (Miltenyi Biotec) according to the manufacturer's protocol. Cells were rinsed with PBS containing 0.5% BSA (Sigma), and the CD326‐positive cells were collected. Then, the CD326‐positive cells were plated onto laminin (2 mg/mL) coated plates with SSC culture medium. The SSC medium was composed of DMEM, 15% foetal bovine serum (FBS, Thermo Fisher Scientific), 50 µmol/L‐mercaptoethanol (Thermo Fisher Scientific), 1 × minimal essential medium (MEM) non‐essential amino acids (Thermo Fisher Scientific), and 10 ng/mL mouse GDNF (Peprotech).

### 2.3 Isolation of Sertoli cells and Leydig cells

Sertoli cells and Leydig cells were isolated from the testes of 21‐day‐old mice. Briefly, the testes denuded of tunica albuginea were incubated with 1 mg/mL collagenase type IV in DMEM (Gibco) for 15 minutes at 37°C on a shaker and then filtered through 100 μm cell strainer (Falcon) to isolate Leydig cells. The filtered seminiferous tubules were further digested with 0.25% trypsin‐EDTA (Gibco) for 15 minutes at 37°C on a shaker and then filtered through 40 μm cell strainer (Falcon). Cells in the filtrate were collected by centrifugation (250 g, 5 minutes) and resuspended in DMEM medium (Gibco) with 10% FBS (Life Technologies). Subsequently, the cell suspensions containing primary Sertoli cells and spermatogonium were cultured in cell culture flasks at 37°C with 5% CO_2_. After 24 hours, the culture supernatant was collected to obtain spermatogonium and the adherent cells were treated with a hypotonic solution (20 mmol/L Tris, pH 7.4) for 2 minutes to obtain pure Sertoli cells. Sertoli cells and Leydig cells were cultured in DMEM supplemented with 10% FBS at 37°C with 5% CO_2_.

### 2.4 Extraction of exosomes from Sertoli cells and Leydig cells

For exosome isolation, equal numbers of Leydig cells and Sertoli cells were transplanted into T75 flasks and maintained in fresh DMEM with 10% Exosome‐depleted FBS Media Supplement (SBI). After 48 hours, culture medium was collected and filtrated through 0.22‐μm filters (Millipore). Exosomes were collected by differential centrifugation. Briefly, the supernatant was centrifuged as follows: 300 g for 10 minutes, 10 000 g for 30 minutes and 100 000 g for 70 minutes (all the steps were performed at 4°C). For exosomes purification, the pellets were followed by an additional washing step with PBS at 160 000 g for 1 hour. Exosomes were resuspended in basal medium and stored at −80°C.

### 2.5 Nanoparticle tracking analysis

Exosomes from Sertoli and Leydig cells were suspended in PBS, respectively, to maintain their concentrations within the measurable concentration range of NanoSight instrument. Each sample was performed for four recordings.

### 2.6 Transmission electron microscope

The obtained exosomes were fixed with fixative buffer containing 2% paraformaldehyde and 2.5% glutaraldehyde in 0.1 mol/L PBS. After embedding, samples were cut into 0.12 μm sections and stained with 0.2% lead citrate and 1% uranyl acetate. The images were photographed under an electron microscope.

### 2.7 PKH26‐labelled exosome transfer

Exosomes from Sertoli cells were labelled using a PKH26 red fluorescent labelling kit (Sigma) according to the manufacturer's protocol. Briefly, the exosomes were incubated with the PKH26 dye for 4 minutes, and the reaction was terminated by adding exosome‐depleted FBS Media Supplement (SBI). Then, the exosomes were washed three times and excess PKH26 dye removed by 100 kD Amicon Ultra‐4 (Millipore), then incubated with SSCs. The rate of uptake of the exosomes into cells was measured by flow cytometry.

### 2.8 Western blotting

After the cell samples were lysed and the protein concentration was determined by BCA. The samples were separated by electrophoresis on 10% SDS‐PAGE gel and transferred to PDVF membrane. After 1 hour of sealing with 5% skimmed milk, PTEN (Abcam), STRA8 (CST) antibodies were added and incubated overnight at 4°C. Membranes were then rinsed six times (7 minutes each) with TBST and incubated with an HRP‐conjugated secondary antibody (1:5000) at room temperature for 1 hour. Membranes were rinsed six times (7 minutes each) with TBST, and immunoreactions were detected by enhanced chemiluminescence (ECL) detection. The protein expression levels were normalized to GAPDH.

### 2.9 Immunofluorescence

Cells were fixed in 4% paraformaldehyde for 30 minutes and washed three times with PBS. The cells were treated in 0.2% Triton‐X for 10 minutes. Non‐specific adhesion sites were blocked with 3% bovine serum albumin (BSA; Sigma, Poole, Dorset, UK) for 30 minutes at room temperature. The primary and secondary antibodies were diluted in a solution of PBS containing 3% BSA, 1% horse serum and 0.1% Triton X‐100. Cells were incubated with primary antibodies SYCP3 (Abcam) overnight at 4°C, followed by incubation with secondary antibodies for 2 hours at room temperature. Nuclei were stained with DAPI (Thermo Fisher Scientific). Stained samples were then visualized, and images were captured using a LSM710 confocal microscope (Zeiss) and analysed by the Image J software.

### 2.10 Total RNA extraction by qRT‐PCR

Total RNA was extracted from exosomes or cells using Trizol (Invitrogen) according to the manufacturer's instructions. Briefly, Trizol (Invitrogen) was added to the exosome or cells precipitation, and homogenized at room temperature for 5 minutes. Then, trichloromethane was added and mixed thoroughly, followed by centrifugation at 12 000 g at 4°C for 15 minutes. The supernatant (upper clear phase) was collected and added with isopropanol, followed by another centrifugation at 12 000 g for 10 minutes at 4°C. The liquid supernatant was discarded, and the pellet was washed with 75% ethanol. The RNA was resuspended in 20 μL RNase‐free DEPC‐water and stored at −80°C.

Reverse transcription reaction for mRNA was conducted using PrimeScript™ RT Master Mix (TAKARA). qPCR was conducted with ChamQ SYBR qPCR Master Mix (Vazyme Biotech) according to the manufacturer's instructions. Signals were visualized using a CFX Connect Real‐Time PCR Detection System (Bio‐Rad). The relative gene expressions levels were normalized to those of *Gapdh*. Quantification was performed via the comparative 2^−ΔΔCt^ method. The primers used are listed in Table 1.

**TABLE 1:** Primer sequences

Gene	Sequence
miR‐486‐5p	F: GCAGTCCTGTACTGAGCTG R: GTCCAGTTTTTTTTTTTTTTTCTCG
U6	F: CTCGCTTCGGCAGCACA R: AACGCTTCACGAATTTGCGT
Stra8	F: GTTTGCCACCTGCAACTCAG R: GGGCTCTGGTTCCTGGTTTA
Sycp3	F: GGGGCCGGACTGTATTTACT R: CTTCCACCAGGCACCATCTT
Gapdh	F: CAGCCTTCCTTCTTGGGTAT R: TGGCATAGAGGTCTTTACGG

Reverse transcription and qRT‐PCR for exosomal miRNA, as well as internal reference U6, were performed using miRNA RT‐PCR Quantitation Kit (Qiagen) according the manufacturer's instructions. Briefly, after an initial denaturation step at 95°C for 3 minutes, the amplifications were carried out with 40 cycles at a melting temperature of 95°C for 15 seconds, and an annealing temperature of 62°C for 34 seconds. The relative expression level of exosomal miRNAs was calculated with the 2^−ΔΔCt^ method. The primers are listed in Table 1.

### 2.11 Cell transfection

miR486‐5p mimic, inhibitor and their corresponding negative controls were all purchased from Genepharma. All cells were seeded in 6‐well plates at a density of 1 × 10^6^ cells/well and grown to 70% in confluence for preparation. Subsequently, 2 μL of 20 μmol/L miRNA mimic stock solution and 3 μL RNAiMAX (Invitrogen, USA) were, respectively, diluted with 250 μL serum‐free medium Opti‐MEM and incubated at room temperature for 5 minutes. Then, the two solutions were mixed and incubated at room temperature for 20 minutes. A total of 500 μL miRNA mimics‐RNAiMAX mixture was added to each well which was supplemented with DMEM complete medium without antibiotics to a total volume of 2 mL. Finally, the culture plates were incubated. After 48 hours, the cell samples were collected. Western blotting was used to detect the expression of related proteins.

### 2.12 Co‐culture experiment

After 24 hours of transfection with miRNA mimic, Sertoli cells (donor cells) were inoculated at a density of 1 × 10^5^/well in the upper chamber of a Transwell (0.4 μm), whereas, one day earlier, the SSCs were seeded into the lower chamber at a density of 1 × 10^5^/well. After incubation for 24 hours, the expression of Cy3‐miR‐486‐5p in the recipient cells was observed under a LSM710 confocal microscope (Zeiss). The expression of miR‐486‐5p in recipient cells was detected by Stem‐loop real‐time RT‐PCR.

### 2.13 Statistical analyses

All experiments were repeated at least three times, and data were expressed as the mean ± one standard deviation around the mean (SD). Statistical analyses were performed by Prism software (GraphPad Software). Statistical analyses were performed with an unpaired Student's t test or one‐way ANOVA for more than two groups. A two‐tailed value of *P* < 0.05 was considered statistically significant.

## 3 RESULTS

### 3.1 Collection and identification of exosomes from Sertoli cells and Leydig cells

**FIGURE 1:**
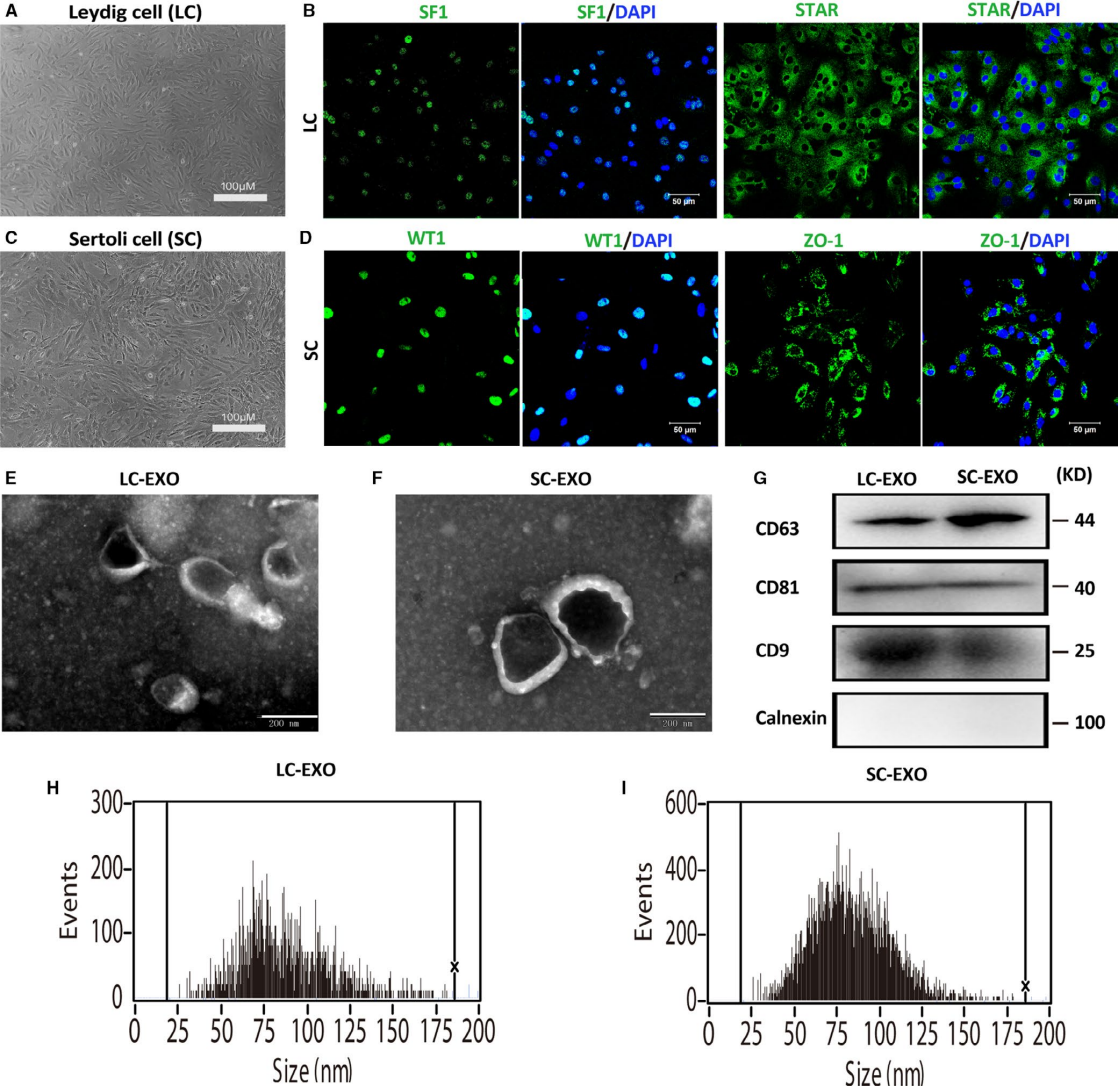
Characterization of SC‐derived exosomes (SC‐EXO) and LC‐derived exosomes (LC‐EXO). A, Representative images of cultured Leydig cells morphology. B, Detection of markers in Leydig cell. The cultured Leydig cells were positive for SF‐1 and StAR. Nuclei were stained with DAPI (blue). Scale bars, 100 μm. C, Representative images of cultured Sertoli cells morphology. D, Detection of markers in Sertoli cells. The cultured Leydig cells were positive for WT1 and ZO‐1. Nuclei were stained with DAPI (blue). Scale bars, 100 μm. E‐F, Exosomes released by Leydig cells (LC‐EXO) and Sertoli cells (SC‐EXO) were detected by electron microscopy. Scale bars, 200 nm. G, The presence of exosomal marker proteins, including CD63, CD9 and CD81, was confirmed by Western blots in SC‐EXO and LC‐EXO. Calnexin was used as negative control. H‐I, Nanoparticle tracking analysis (NTA) demonstrates the size distribution of LC‐EXO and SC‐EXO

Leydig cell (LC) and Sertoli cell (SC) were isolated and purified from puberty (3 weeks post‐partum) (Figure 1A,C). Leydig cell marker (SF1 and STAR) and Sertoli cell marker (WT1 and ZO‐1) were detected in the isolated LCs and SCs (Figure 1B,D). Exosomes were isolated from SC condition medium and LC condition medium, respectively. The purified SC‐derived exosomes (SC‐EXO) and LC‐derived exosomes (LC‐EXO) were observed under a TEM. The cup‐shaped membrane‐bound vesicles with an approximately 100 nm diameter were identified (Figure 1E,F). Additionally, the expression of exosomal markers, including CD63, CD9 and CD81, was detected in SC‐EXO and LC‐EXO, whereas calnexin, an integral protein not expressed in exosomes, was barely detected (Figure 1G). The size range and concentration of the particles were measured by nanoparticle tracking analysis (NTA). The diameters of almost all particles were between 50 and 100 nm. The mean diameter was 84.2 nm in LC‐EXO and 82.2 nm in SC‐EXO (Figure 1H,I). These data demonstrated that the isolated particles were exosomes.

### 3.2 SC‐derived exosomes promote SSC differentiation

**FIGURE 2:**
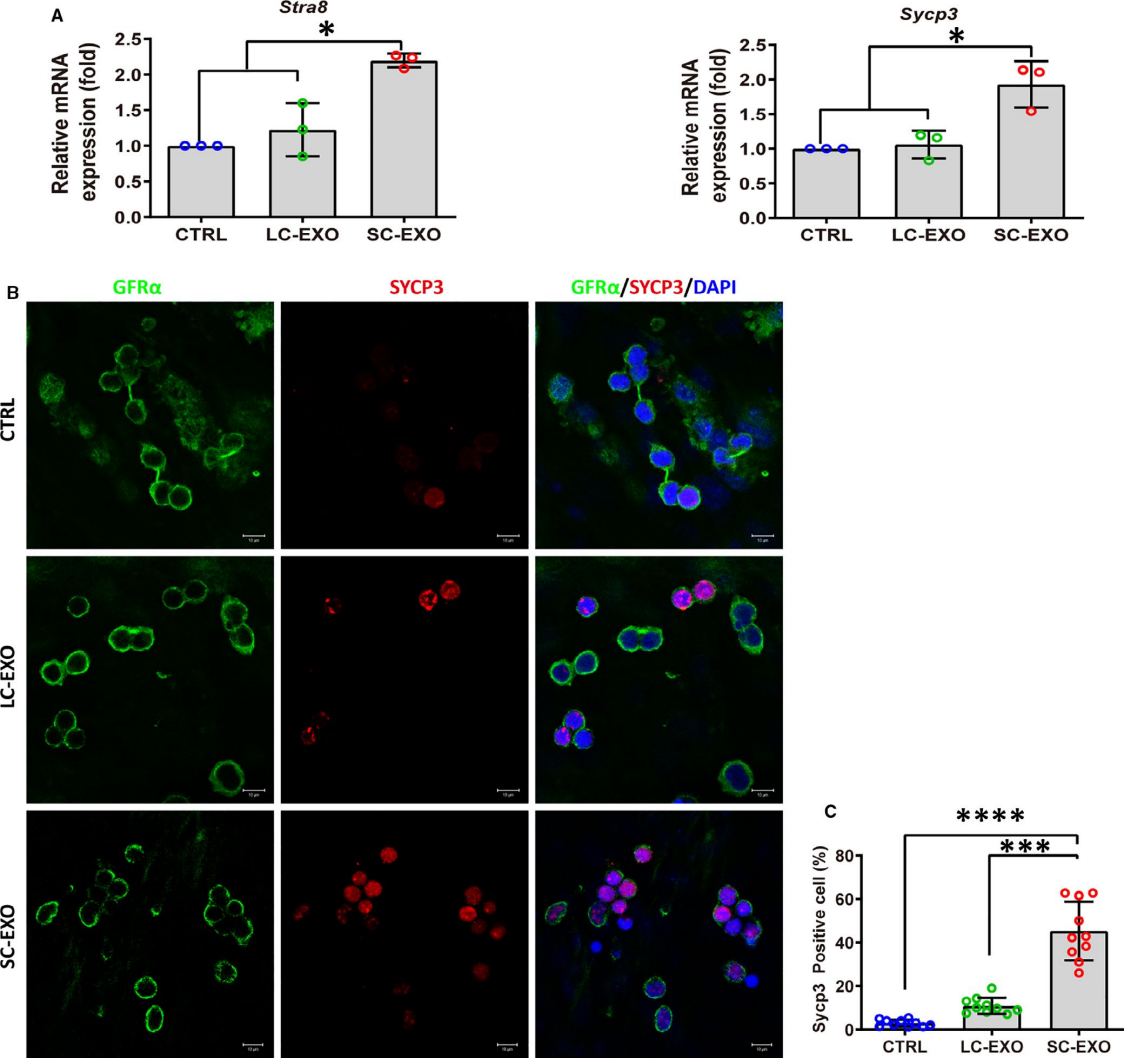
SC‐derived exosomes up‐regulate spermatogonial differentiation markers in SSCs. A, RT‐qPCR determined the expression of genes involved in differentiation of spermatogonia (*Stra8* and *Sycp3*) in SSCs treated with SC‐derived exosomes (SC‐EXO) and LC‐derived exosomes (LC‐EXO) for 72 h. SSCs without exosome treatment were used as control. The copy number of mRNA of each gene was normalized with *Gapdh*, and the data were obtained from three independent experiments and are presented as mean ± SD. *P < 0.05. B, Co‐immunostaining of SSC marker GFRα and meiosis marker SYCP3 in SSCs treated with SC‐EXO and LC‐EXO for 72 h. SSCs without exosome treatment were used as control. Nuclei were stained with DAPI (blue). Scale bars, 10 μm. C, Quantitative analysis of the percentage of SYCP3‐positive cells in (B). Each data point indicates the percentage of SYCP3‐positive cells. Each data point indicates the percentage of SYCP3‐positive cells in one separate image. These images were obtained from three independent experiments, and data were presented as mean ± SD, ***P < 0.001; ****P < 0.0001

We examined whether LC‐EXO and SC‐EXO influence differentiation of SSC. SSCs were plated onto laminin‐coated feeder‐free plates, and treated with SC‐derived exosomes (SC‐EXO) and LC‐derived exosomes (LC‐EXO). After 72 hours of treatment, expression of genes involved in spermatogonial differentiation *Stra8* and *Sycp3* was significantly elevated by SC‐EXO, while LC‐EXO had no obvious effect on expression of *Stra8* and *Sycp3* (Figure 2A). Immunofluorescence results revealed that the percentage of SYCP3‐positive cells was 45.3% in the SC‐EXO treatment group, which was significantly higher than in the LC‐EXO (Figure 2B‐C). These results suggested that SC‐EXO promotes differentiation of SSC.

### 3.3 SC‐Exosome labelling and uptake by SSC

**FIGURE 3:**
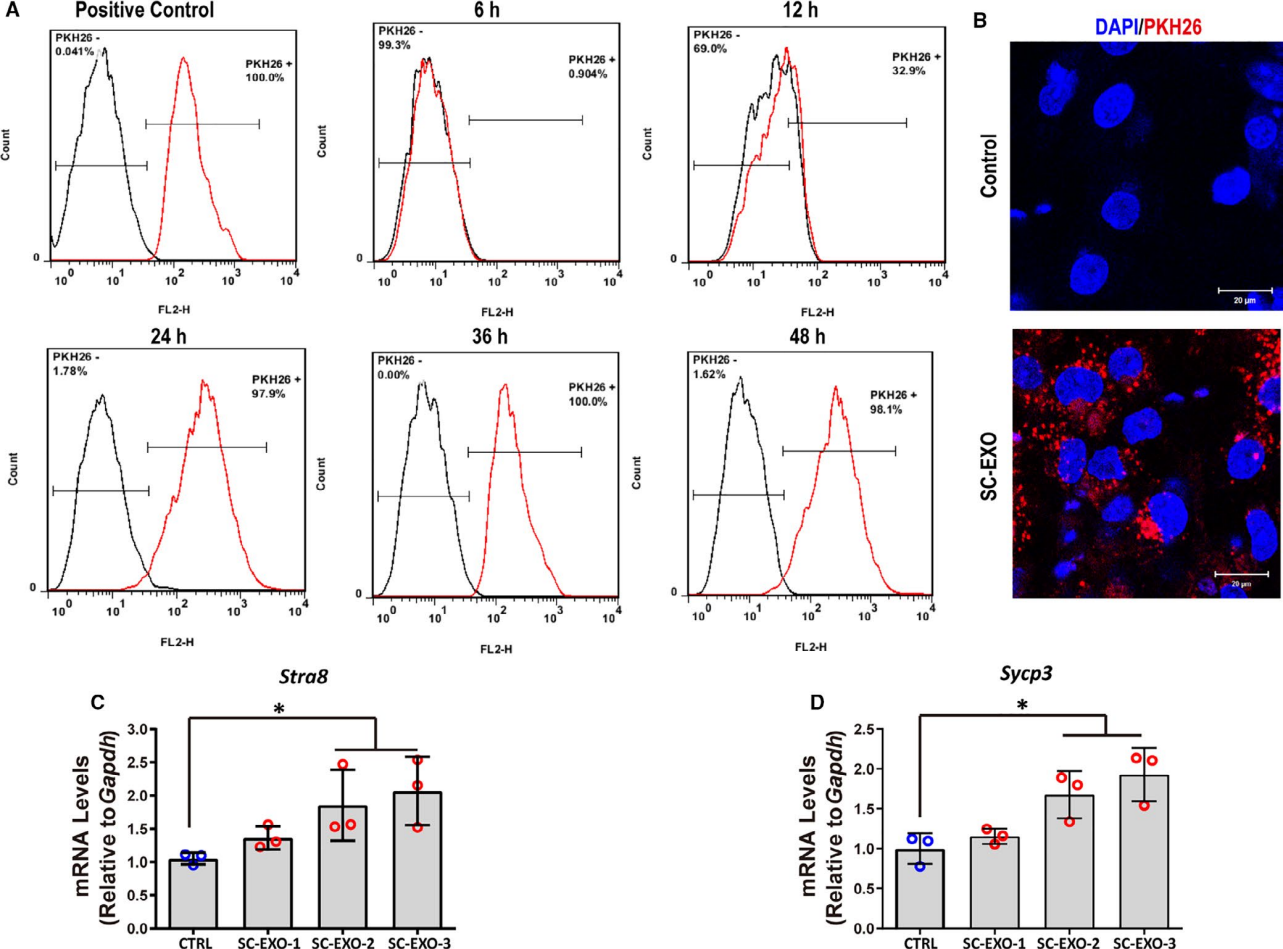
SC‐EXO could be uptake by SSCs and up‐regulate *Stra8* and *Sycp3* expression. A, Flow cytometric analysis of the uptake of SC‐derived exosomes (SC‐EXO) labelled with PKH26 at various times points. SSCs were treated with SC‐EXO labelled with PKH26 (8.88 × 10^9^) for 6, 12, 24, 36 and 48 h. SC‐derived exosomes (SC‐EXO) labelled with PKH26 were completely uptaken by SSCs after 24 h. SSCs labelled with PKH26 were used as positive control. B, Representative fluorescent confocal images of SSCs that were exposed to PKH26‐labelled exosomes (red) from Sertoli cells for 24 h. Nuclei were stained with DAPI (blue). Scale bars, 20 μm. C‐D, RT‐qPCR analysis of *Stra8* and *Sycp3* expression in SSCs treated with SC‐EXO at different concentrations for 24 h. SSCs without exosome treatment were used as control. EXO‐1:4.44 × 10^9^ particles/mL, EXO‐2:8.88 × 10^9^, EXO‐3:1.33 × 10^10^ particles/mL. The copy number of mRNA of each gene was normalized with *Gapdh*, and the data were obtained from three independent experiments and are presented as mean ± SD; *P < 0.05

To confirm whether SC‐EXO could be taken up by SSC, followed promoting SSC differentiation. SC‐EXO were labelled with the PKH26 dye. Then, SSCs were incubated with the PKH26‐labelled SC‐EXO, the efficiency of cellular uptake was analysed by flow cytometry. The uptake efficiency of exosomes increased with incubation time. After 24 hours of incubation, the uptake efficiency of exosomes was 100% (Figure 3A). PKH26‐labelled exosomes exhibited red fluorescence in the cytoplasm of SSC. In contrast, the negative control (NC) group only exhibited a weak red fluorescence signal (Figure 3B). Besides, mRNA levels of *Stra8* and *Sycp3* were significantly up‐regulated in a dose‐dependent manner when SSCs were treated with different concentrations of SC‐EXO for 24 hours (Figure 3C‐D). These data suggested SC‐EXO could be engulfed by SSCs, and promote SSC differentiation.

### 3.4 Exosomes mediate the transfer of miR‐486 from SC to SSCs

**FIGURE 4:**
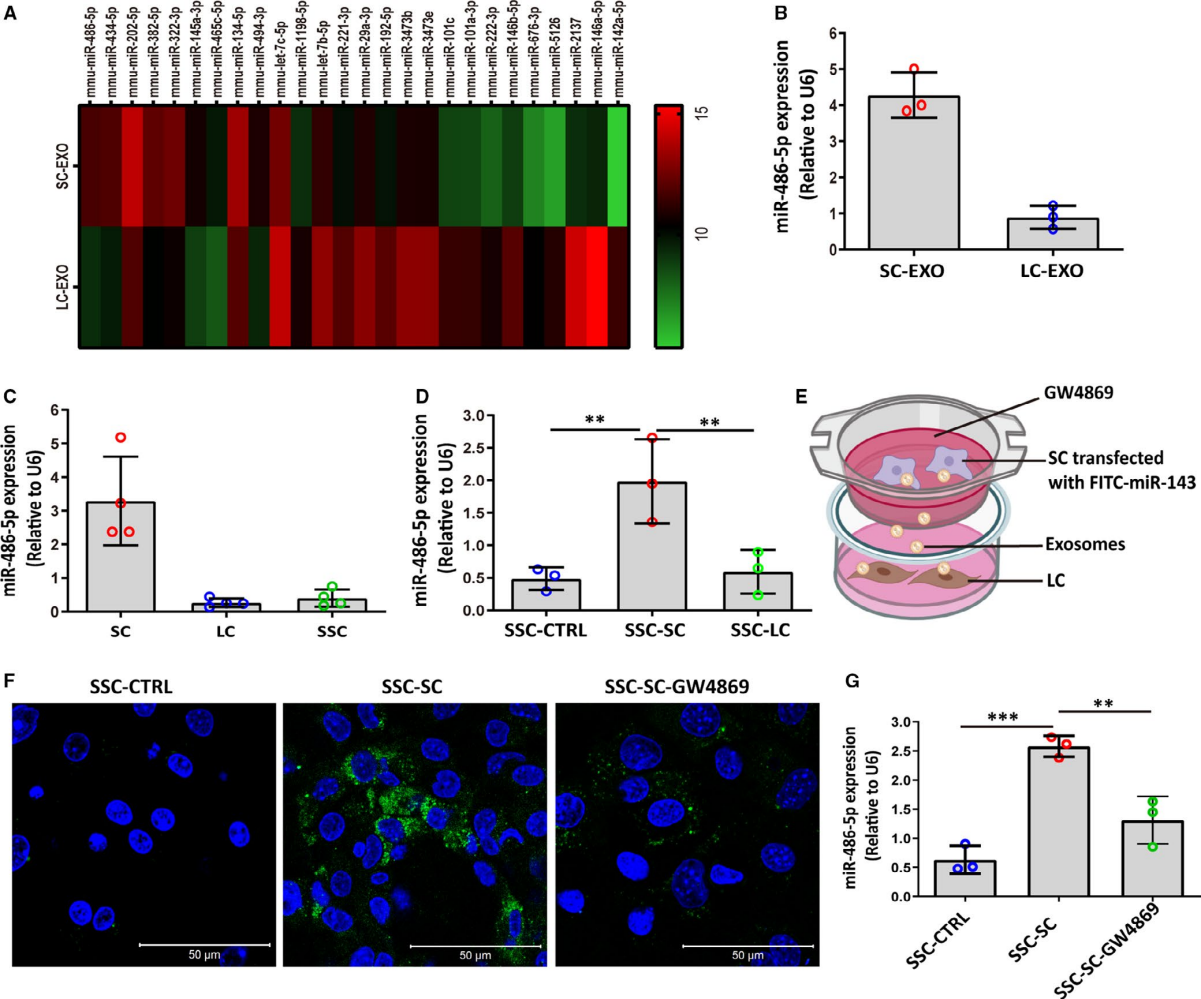
Exosome‐mediated transfer of miR‐486‐5p from SC to SSC. A, Heat map of differential miRNAs between Sertoli cell‐derived exosomes (SC‐EXO) and Leydig cell‐derived exosomes (LC‐EXO). The colour bar indicates miRNA expression in log2 scale. Red and green indicate up‐regulated and down‐regulated miRNAs, respectively. B‐C, RT‐qPCR analysis of miR‐486‐5p expression in LC‐EXO, SC‐EXO, Leydig cell (LC), Sertoli cell (SC). D, Levels of miR‐486 were analysed in SSCs incubated with conditioned medium from SC for 24 h. SSC‐CTRL: SSCs were treated with control medium; SSC‐SC: SSCs were treated with SC conditioned medium; SSC‐LC: SSCs were treated with LC conditioned medium. E, Schematic diagram of co‐culture of SSCs with SCs transfected by FITC‐labelled miR‐486. SCs transfected with FITC‐labelled‐miR‐486 were placed in the upper chamber of transwell; SSCs were seeded in the lower chamber. F, Representative fluorescence images of FITC‐labelled miR‐486 (green) in SSC after co‐culture with SC for 24 h. SSC‐SC: SSCs co‐cultured with SCs transfected by FITC‐labelled miR‐486 for 24 h. SSC‐SC‐GW4869: The SC was transfected with FITC‐labelled miR‐486, followed by treatment with 10 μmol/L GW4869, an inhibitor of neutral sphingomyelinase. G, Expression of miR‐486 was measured in SSC by qPCR after co‐cultured with SCs for 24 h. SSC‐SC: SSCs co‐cultured with FITC‐labelled miR‐486‐transfected SCs for 24 h. SSC‐SC‐GW4869: The SC was transfected with FITC‐labelled miR‐486, followed by treatment with 10 μmol/L GW4869, an inhibitor of neutral sphingomyelinase. The copy number of miR‐486‐5p was normalized with U6, and the data were obtained from three independent experiments and are presented as mean ± SD. **P < 0.01, ***P < 0.001

To further understand the mechanism of SC‐EXO in promoting SSC differentiation, the expression of miRNAs in both SC‐EXO and LC‐EXO was determined. According to the differential analysis, we found miR‐486‐5p was one of the most prominently up‐regulated miRNAs in SC‐EXO compared with LC‐EXO (Figure 4A). qRT‐PCR further revealed that the expression of miR‐486‐5p in SC‐EXO was significantly higher than in LC‐EXO (Figure 4B). In addition, the expression level of miR‐486‐5p was also significantly up‐regulated in Sertoli cells compared to Leydig cells. In contrast, the expression of miR‐486‐5p in SSC was very low (Figure 4C). Interestingly, when the SSC was treated with conditioned medium from LC or SC for 48 hours, the conditioned medium from SC elevated the miR‐486 expression in SSC (Figure 4D). To assess whether SC‐EXO transport miR‐486 to SSC, SCs transfected with fluorescein isothiocyanate (FITC)‐labelled miR‐486 were placed in the upper chamber of a transwell co‐culture system, and SSCs were seeded in the lower chamber (Figure 4E). After 24 hours of co‐culture, SSCs were surrounded by fluorescently labelled miR‐486 mimics. However, the pharmacological inhibition of sphingomyelinase GW4869, which is known to inhibit exosome generation, attenuated the transfer of FITC‐miR‐486 to SSC (Figure 4F), indicating that the miR‐486‐5p transfer was mediated by exosomes. qRT‐PCR analysis further confirmed that the expression of miR‐486 in SSC was significant increase when SSCs were co‐cultured with SCs, while this effect was blunted when SCs were treated with GW4869 (Figure 4G). These findings suggested that SC‐derived exosomes were able to transport miR‐486‐5p to SSC.

### 3.5 miR‐486 directly targets Pten and promotes SSC differentiation

**FIGURE 5:**
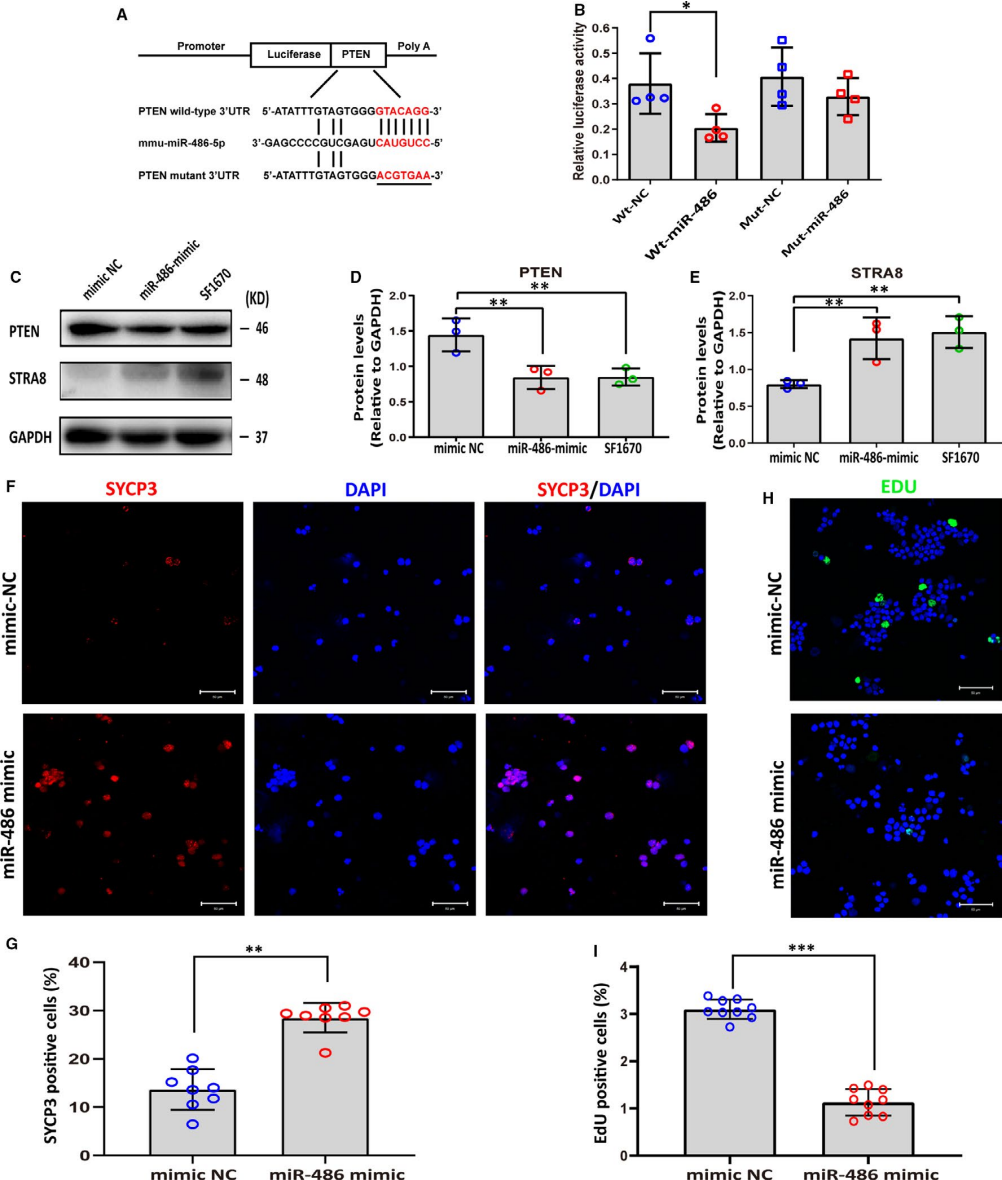
miR‐486‐5p promotes SSC differentiation by targeting Pten. A, The recombinant plasmid (psiCHECK2) with the 3’UTR sequences of Pten containing the predicted binding site sequence (wild‐type, Wt) of miR‐486‐5p, and nucleotides mutated (Mut) in 3’UTR sequences of Pten. B, Luciferase reporter assay showed the miR‐486‐5p mimic reduced luciferase activity in 293T cells with wild‐type Pten, but not the mutant. C‐E, Transfection of miR‐486‐5p mimic decreased the expression of PTEN and up‐regulated STRA8 expression in SSCs. SSCs treated with PTEN inhibitor SF1670 were used as positive control. (C) Representative Western blot image. (D‐E) Quantitative results of PTEN and STRA8 protein levels in SSCs transfected with miR‐486‐5p mimic. GAPDH served as a loading control and the data were obtained from three independent experiments and are presented as mean ± SD; *P < 0.05; **P < 0.01. F, Detection of SYCP3 (red) expression in SSCs transfected with miR‐486‐5p mimic or miR‐486‐5p mimic‐NC by immunofluorescence. Nuclei were stained with DAPI (blue). Scale bars, 50 μm. G, Summary of SYCP3‐positive cells in (F). Each data point indicates the percentage of SYCP3‐positive cells in one separate image. These images were obtained from three independent experiments, and data were presented as mean ± SD, **P < 0.01. H, EdU incorporation assay showed the EdU‐positive cells in SSCs transfected with miR‐486‐5p mimic or mimic NC. Nuclei were counterstained with DAPI. Scale bars, 50 μm. I, Summary of EdU‐positive cells in (H). EdU‐positive (green) rate was remarkably decreased when miR‐486‐5p was overexpressed in SSCs. The percentages of EdU‐positive cells were counted out of 500 total cells from independent ten images. These images were obtained from three independent experiments, and data were presented as mean ± SD, ***P < 0.001

TargetScan was applied to predict the possible target genes. Among hundreds of genes that were predicted as potential targets, Pten was selected. To test whether Pten is a direct target of miR‐486‐5p, the recombinant plasmids (psiCHECK2) with the 3’UTR sequences of Pten containing the predicted binding site sequence (wild‐type, wt) of miR‐486‐5p were constructed for luciferase assays. And recombinant plasmids containing the Pten 3’UTR sequence with mutant nucleotides (mutant type, mut) were also constructed to serve as control (Figure 5A). The Wt and mut vectors transfected into HEK293T cells with miR‐486 mimic or mimic NC, and the level of luciferase enzyme activity was measured. Overexpression of miR‐486 suppressed the luciferase activity of the reporter gene, whereas mutation of miR‐486 binding sites abolished this repression of luciferase activity (Figure 5B), which confirmed that Pten is a target gene of miR‐486‐5p. miR‐486‐5p binds to the Pten mRNA 3’‐UTR. Moreover, it was found that miR‐486‐5p mimic down‐regulated the protein levels of PTEN when miR‐486‐5p mimic was transfected into SSCs. To verify the role of miR‐486‐5p in the differentiation of SSC, the expression level of STRA8 was determined. Result showed that miR‐486‐5p up‐regulated the expression of STRA8 significantly. In addition, SSCs were treated with PTEN inhibitor SF1670 to examine whether it regulates the expression of STRA8. SF1670 significantly down‐regulated expression of PTEN compared to the control, while the level of STRA8 was significantly elevated (Figure 5C‐E). These results suggested that miR‐486‐5p increased STRA8 expression level by targeting PTEN in SSC.

STRA8 plays a key role in the initiation of meiosis in mammals. Immunofluorescence revealed that miR‐486‐5p mimic increased the percentages of SYCP3‐positive cell from 13.6% to 28.5% (Figure 5F,G). In addition, EdU incorporation assays showed that the EdU‐positive rate was remarkably decreased when miR‐486‐5p was overexpressed in SSC, suggesting that miR‐486‐5p inhibited SSC proliferation (Figure 5H,I). Collectively, these results demonstrated that miR‐486‐5p inhibited the self‐renewal of SSC and promoted SSC differentiation by targeting PTEN.

### 3.6 SC‐derived miR‐486 transfer from SC to SSC promotes differentiation of SSC through the PTEN/STRA8 axis

**FIGURE 6:**
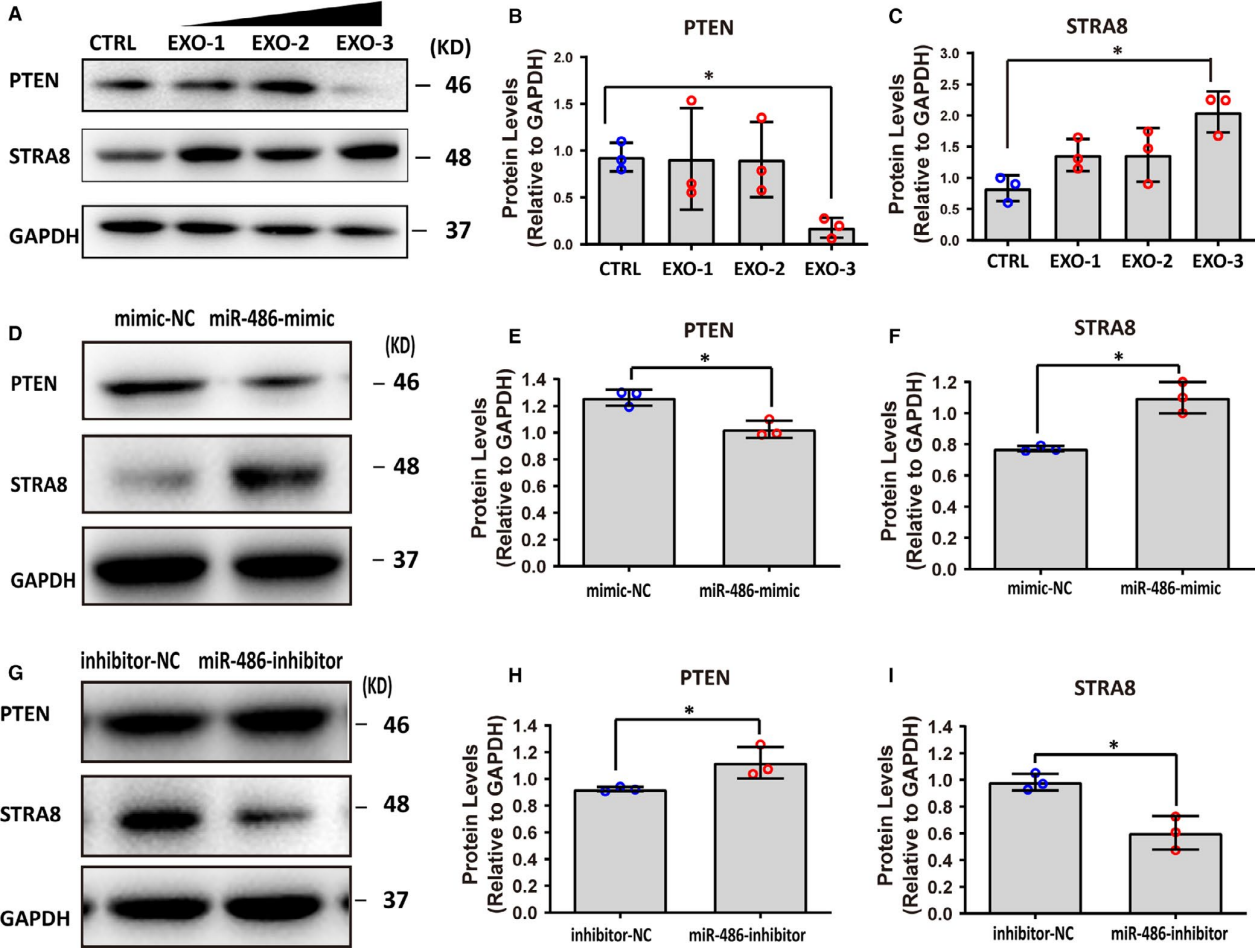
miR‐486‐5p derived from SCs promoted differentiation of SSC through the PTEN/STRA8 axis. A‐C, SC‐EXO suppressed expression of PTEN and up‐regulated STRA8 expression in SSCs. SSCs treated with SC‐EXO at defined concentrations for 48 h. EXO‐1: 4.44 × 109 particles/mL, EXO‐2: 8.88 × 109, EXO‐3: 1.33 × 1010 particles/mL. A, Representative Western blot image. (B‐C) Summary of the protein levels of PTEN and STRA8 in (A). GAPDH served as a loading control. Data points were obtained from three independent experiments and were presented as mean ± SD, *P < 0.05. D‐F, Western blot analysis of PTEN and STRA8 in SSCs co‐cultured with SC transfected with miR‐486 mimic or NC for 48 h. D, Representative Western blot image. (E‐F) Summary of the protein levels of PTEN and STRA8 in (D). GAPDH served as a loading control. mimic‐NC: SCs were transfected with miR‐486 mimic NC; miR‐486‐mimic: SCs were transfected with miR‐486 mimic. Data points were obtained from three independent experiments and were presented as mean ± SD, *P < 0.05. G‐I, Western blot analysis of PTEN and STRA8 in SSCs co‐cultured with SC transfected with miR‐486 inhibitor or NC for 48 h. G, Representative Western blot image. (H‐I) Summary of the protein levels of PTEN and STRA8 in (D). GAPDH served as a loading control. inhibitor‐NC: SCs were transfected with miR‐486 inhibitor NC; miR‐486 inhibitor: SCs were transfected with miR‐486 inhibitor. Data points were obtained from three independent experiments and were presented as mean ± SD, *P < 0.05

Spermatogonial stem cell were treated with different concentrations of SC‐EXO to assess whether miR‐486‐5p were packaged into exosomes and down‐regulated PTEN expression. In line with above findings, SC‐EXO at a concentration of 1.3 x10^10^ particles/mL has been shown to suppress the expression of PTEN and increase the expression of STRA8 (Figure 6A‐C).

**FIGURE 7:**
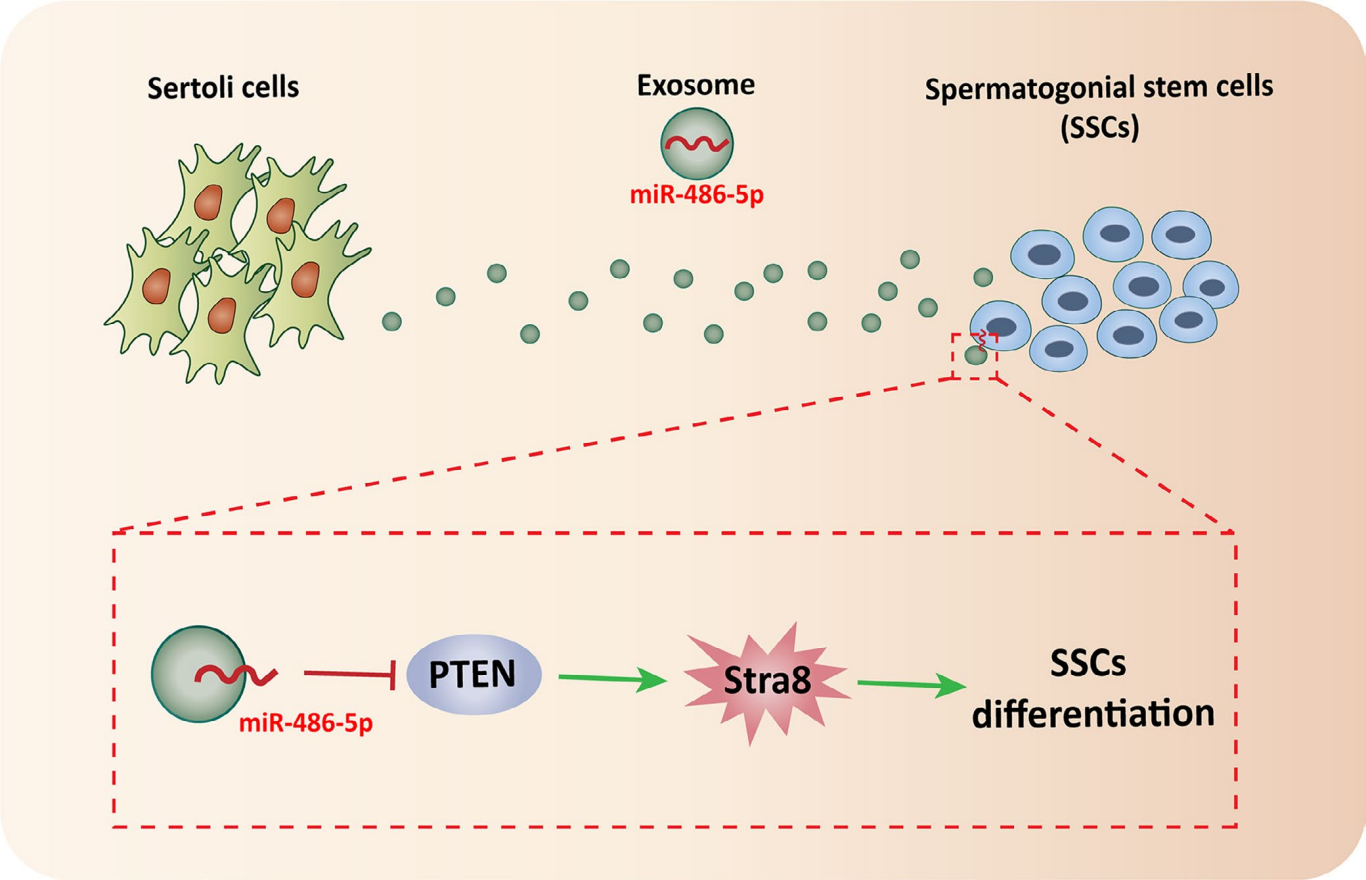
Schematic diagram of Sertoli cell‐derived exosome miR‐486‐5p promotes spermatogonial stem cell differentiation by targeting PTEN

To further detect the effect of miR‐486‐rich exosomes on differentiation of SSC, co‐culture experiment was conducted, in which SC was transfected with either a miR‐486 mimic or anti‐miR‐486. The expression of PTEN in SSC was determined after 48 hours of co‐culture. As expected, overexpression of miR‐486 in SC significantly down‐regulated PTEN expression and elevated STRA8 expression (Figure 6D‐F). In contrast, anti‐miR‐486 transfection up‐regulated the expression of PTEN and decreased STRA8 expression (Figure 6G‐I). Collectively, these results indicated that SC‐derived miR‐486‐5p was transported to SSC by exosomes, promoting SSC differentiation via PTEN/STRA8 (Figure 7).

## 4 DISCUSSION

In testis, Sertoli cell provides an appropriate microenvironment or niche for the development of male germ cells, which is indispensable for maintaining normal spermatogenesis. Therefore, it is necessary to determine the potential molecular mechanisms of Sertoli cell during spermatogenesis, which will offer novel insights into the aetiology of infertility and provide new targets for gene therapy in male infertility. Exosomes are critical components in the crosstalk between cells. The biological role of exosomes on target cells depends on the selectively packages of miRNA content.^19^ In this study, we demonstrated that Sertoli cell‐secreted miR‐486‐containing exosomes can transfer from Sertoli cell to SSC, and promote SSC differentiation through direct targeting PTEN.

We selected miR‐486 for further functional studies due to its up‐regulated expression in SC‐derived exosomes compared to LC‐derived exosomes. Furthermore, miR‐486‐5p was enriched in SC causing the active packaging of miR‐486‐5p into exosomes. To demonstrate whether miR‐486‐5p was packaged into exosomes, the SSC was treated with conditioned medium from LC or SC for 48 hours. The SC conditioned medium elevated the miR‐486 expression in SSC. Besides, miR‐486‐5p in SSC was significantly up‐regulated when SSC co‐cultured with SC transfected with miR‐486‐5p. In contrast, anti‐miR‐486‐5p in SC attenuated the transfer of miR‐486‐5p to SSC. These data indicated that the SC‐derived exosomes up‐regulate miR‐486‐5p expression in SSC.

Previous studies proved that miR‐486‐5p regulated the proliferation and differentiation of various types of cells.^20^, ^21^, ^22^ Also, overexpression of miR‐486‐5p promotes differentiation and suppresses apoptosis in myeloid cells.^23^ Exosome‐derived miR‐486‐5p regulates cell cycle and inhibits cell proliferation in lung adenocarcinoma.^24^ Additionally, exosome‐derived miR‐486‐5p is necessary for the development of the cow's mammary gland.^17^ However, the role of miR‐486‐5p in SSC remains poorly studied. Therefore, we explored the effects of miR‐486‐5p on the differentiation of SSC.

miRNAs exert their biological functions by degrading or inhibiting their target mRNAs. Our study demonstrated miR‐486‐5p targeted *Pten*. Overexpression of miR‐486‐5p down‐regulated the expression of PTEN and up‐regulated the expression of STRA8. STRA8 is a vertebrate‐specific, cytoplasmic factor expressed by germ cells in response to retinoic acid, which is a vital gatekeeper of meiotic initiation.^25^ In male germline, meiosis is initiated through the induction of STRA8 by retinoic acid. RA induce STRA8 expression, which promote SSC differentiation.^25^ *Stra8*‐deficient germ cell in post‐natal males arrest just before meiosis, without entering meiotic prophase.^26^ However, it remains to be elucidated whether the other genes also regulate *Stra8* and promote SSC differentiation. In this study, we found miR‐486‐5p targeted PTEN to increase *Stra8* expression and promote SSC differentiation.

Phosphatase and tensin homolog deleted on chromosome ten (PTEN) is a tumour suppressor, which classically counteracts the PI3K/AKT/mTOR signalling cascade.^27^ It governs a lot of cellular processes including survival, proliferation, energy metabolism and cellular architecture.^28^ High susceptibility of PTEN gene to mutation and loss of its normal function is frequently found in a variety of cancers.^29^ Additionally, nuclear PTEN also plays an important role in chromosome stability, DNA repair and apoptosis by phosphatase‐independent tumour suppressive functions.^30^ In reproductive system, the PTEN/PI3K/Akt is a major signal pathway governing primordial follicle recruitment and growth, the size of the primordial follicle pool is determined by the dynamic activity of this pathway.^31^ In normal testis, PTEN was abundant in spermatogonia, present in spermatocytes and spermatids, while it was not detectable in spermatozoay,^32^ which indicated PTEN negatively correlated with the differentiation of spermatogonia. Additionally, among hundreds of genes predicted as potential targets of miR‐486‐5p, PTEN was expressed in SSC. Therefore, PTEN was selected as potential target of miR‐486‐5p. Our study suggested that miR‐486‐5p promoted differentiation of SSC by targeting the 3’UTRs of PTEN. The decline of PTEN expression up‐regulated STRA8 expression. The inhibitor of PTEN increased the expression of STRA8, which further confirmed that PTEN regulated the expression of STRA8. However, the molecular mechanism of how PTEN modulate STRA8 expression has yet to be elucidated. Future studies will be directed towards understanding the mechanisms for how PTEN negatively regulated SSC differentiation. Insights into the role of PTEN in SSC differentiation will inform the rational design of novel therapies for infertility.

In conclusion, our findings shed light on that miR‐486‐containing exosomes mediated SC‐SSC crosstalk. miR‐486‐5p transported by exosomes promoted the differentiation of SSC via targeting PTEN, thereby providing a mechanism on how miRNA contributes to regulating the differentiation of male germ cells. SC‐derived miR‐486 can be utilized as a potential biomarker for the diagnosis of male infertility.

## CONFLICT OF INTEREST

The authors confirm that there are no conflicts of interest.

## AUTHOR CONTRIBUTIONS

**Quan Li:** Formal analysis (equal); Investigation (equal); Writing‐review & editing (equal). **Hanhao Li:** Data curation (equal); Writing‐review & editing (equal). **Jinlian Liang:** Formal analysis (equal); Investigation (equal); Writing‐review & editing (equal). **Jiaxin Mei:** Data curation (equal); Writing‐review & editing (equal). **Cao Zhen:** Writing‐review & editing (equal). **Lei Zhang:** Data curation (equal); Writing‐review & editing (equal). **Jiao Luo:** Data curation (equal); Writing‐review & editing (equal). **Yan Tang:** Data curation (equal); Writing‐review & editing (equal). **Rufei Huang:** Data curation (equal); Writing‐review & editing (equal). **Huan Xia:** Data curation (equal); Writing‐review & editing (equal). **Qihao Zhang:** Data curation (equal); Writing‐review & editing (equal). **Qi Xiang:** Data curation (equal); Writing‐review & editing (equal). **Yan Yang:** Methodology (equal); Supervision (equal); Writing‐original draft (equal); Writing‐review & editing (equal). **Yadong Huang:** Funding acquisition (lead); Methodology (equal); Project administration (lead); Supervision (equal); Writing‐original draft (equal); Writing‐review & editing (equal).

## Data Availability

The data that support the findings of this study are available from the corresponding author upon reasonable request.
